# Miniaturized Salting-Out Assisted Liquid-Liquid Extraction Combined with Disposable Pipette Extraction for Fast Sample Preparation of Neonicotinoid Pesticides in Bee Pollen

**DOI:** 10.3390/molecules25235703

**Published:** 2020-12-03

**Authors:** Xijuan Tu, Wenbin Chen

**Affiliations:** 1 College of Bee Science, Fujian Agriculture and Forestry University, Fuzhou 350002, China; xjtu@fafu.edu.cn; 2 College of Animal Science, Fujian Agriculture and Forestry University, Fuzhou 350002, China

**Keywords:** sample preparation, disposable pipette extraction, neonicotinoid pesticides, bee pollen, HPLC, salting-out assisted liquid-liquid extraction

## Abstract

As the main source of nutrients for the important pollinator honeybee, bee pollen is crucial for the health of the honeybee and the agro-ecosystem. In the present study, a new sample preparation procedure has been developed for the determination of neonicotinoid pesticides in bee pollen. The neonicotinoid pesticides were extracted using miniaturized salting-out assisted liquid-liquid extraction (mini-SALLE), followed by disposable pipette extraction (DPX) for the clean-up of analytes. Effects of DPX parameters on the clean-up performance were systematically investigated, including sorbent types (PSA, C18, and silica gel), mass of sorbent, loading modes, and elution conditions. In addition, the clean-up effect of classical dispersive solid-phase extraction (d-SPE) was compared with that of the DPX method. Results indicated that PSA-based DPX showed excellent clean-up ability for the high performance liquid chromatography (HPLC) analysis of neonicotinoid pesticides in bee pollen. The proposed DPX method was fully validated and demonstrated to provide the advantage of simple and rapid clean-up with low consumption of solvent. This is the first report of DPX method applied in bee pollen matrix, and would be valuable for the development of a fast sample preparation method for this challenging and important matrix.

## 1. Introduction

As the most important managed pollinator, the honeybee is crucial to the ecosystem, agriculture, and food production. However, precipitous loss of the honeybee population has been reported in Europe and North America, which raises the concern of a pollination crisis [1]. Multiple stressors have been considered as potential causes of the honeybee decline, including nutrition, pesticide, parasites, and disease [2].

Bee pollen is the major source of protein in the honeybee diet, and it also provides the honeybee with essential nutrients, e.g., lipids, vitamins, and minerals [3]. In addition to nutritional values, phytochemicals in bee pollen were reported to be critical in the up-regulating detoxification and immunity genes of the western honeybee [4]. Thus, the security of bee pollen is important for keeping the honeybee healthy. However, bee pollen has the potential to be contaminated by pesticides due to the widespread use of chemicals in plant protection.

Determination of pesticide residues in bee pollen is important for evaluating the risk of exposure. Because of the complexity of the constituents in bee pollen, sample preparation to extract and clean up target compounds is generally required when chromatography-based technology is employed for the analysis of pesticide residues [5]. Classical solid-liquid extraction [6], solid-phase extraction (SPE) [7,8], matrix solid-phase dispersion (MSPD) [9], and dispersive solid-phase extraction (d-SPE) [10,11,12] methods have been developed for the determination of pesticides in bee pollen. Despite this progress, it is still a great challenge to develop a simple and rapid sample preparation method for residue analysis in bee pollen matrix. In the present work, we have proposed a new sample preparation procedure based on salting-out assisted liquid-liquid extraction [13] and disposable pipette extraction (DPX) clean-up for the determination of neonicotinoid residues in bee pollen.

DPX is an alternative solid-phase extraction (SPE) method which demonstrates the advantages of reducing labor, time, and solvent consumption in the clean-up of analytes [14,15]. In a typical DPX device, sorbents are assembled in a pipette tip with a screen at the bottom and a barrier on the top [16]. Sample solution may be introduced by drawing in from the bottom of the tip or loading on the top of it [16,17]. The analytes are then washed and eluted from the sorbent with a suitable solvent. The operation of DPX is simple and labor-saving. In addition, it allows for automated and high-throughput preparation when combined with the liquid handling system [18,19]. This sample preparation method has been widely used in the analysis of drugs [14,18,19,20,21,22,23,24], pesticide residues [16,17,25,26,27,28,29,30], heavy metal ions [31], and environmental contaminants [32,33,34,35]. In the present work, the parameters of DPX were systematically investigated on the clean-up of neonicotinoid pesticides in bee pollen. To the best of our knowledge, this is the first report on the use of the DPX method for the matrix of bee pollen.

## 2. Results and Discussion

### 2.1. Salting-Out Assisted Liquid-Liquid Extraction

A miniaturized salting-out assisted liquid-liquid extraction (mini-SALLE) was used for the rapid extraction of three neonicotinoid pesticides—thiamethoxam, acetamiprid, and thiacloprid—from bee pollen (Figure S1). In the mini-SALLE protocol, 2 mL ACN mixed with 2 mL H_2_O was applied as the extraction solution. The bee pollen sample was homogenized with the ACN-H_2_O solution before phase separation agent was introduced to trigger the partition of ACN from the mixture. Salts, 0.4 g of MgSO_4_ and 0.2 g of NaCl, were used to induce phase separation and extract target compounds into the upper ACN phase. The former, MgSO_4_, has been demonstrated to be an efficient phase-separation agent as the high extraction yield for relatively high-polarity compounds [36]. The latter, NaCl, was used to reduce the co-extracted protein and sugar contents from the matrix [37,38].

**Figure 1:**
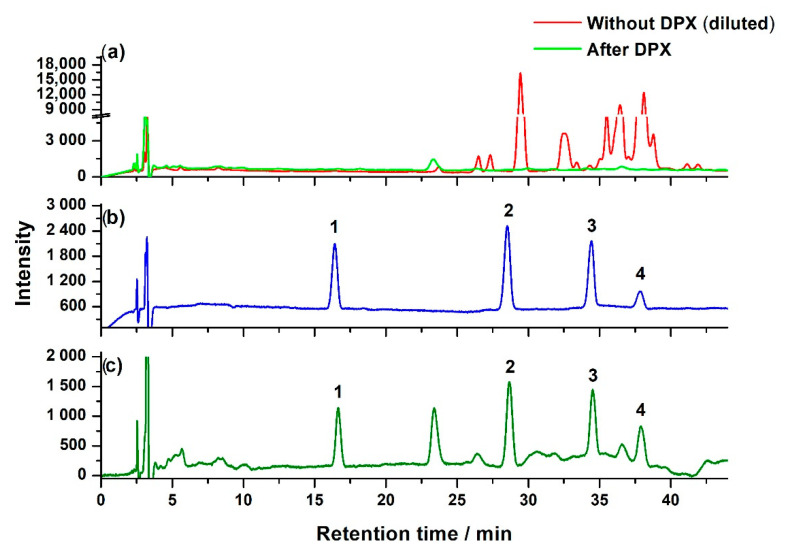
Representative chromatogram of (**a**) diluted SALLE extract without DPX and SALLE extract after PSA-based DPX, (**b**) standards of neonicotinoid pesticides and internal standard, (**c**) spiked bee pollen sample after SALLE and following PSA-based DPX clean-up. Peak 1: thiamethoxam, 2: acetamiprid, 3: thiacloprid, and 4: ethyl 6-chloropyridine-2-carboxylate (internal standard).

The obtained SALLE extract from the bee pollen showed a high background of matrix interferences. Bee pollen is a complex matrix which contains pigments, nutrients such as proteins, carbohydrates, vitamins, lipids, and various phytochemical compounds [3,39,40]. We observed that the final extract after SALLE (upper ACN phase) was still a light yellow color, though the phase partition had retained a substantial number of matrix compounds in the lower H_2_O phase. Moreover, as shown in Figure 1a, the diluted SALLE extract showed complicated and high-intensity chromatography peaks. It is important to note that the SALLE extract was diluted 50 times before being injected into the HPLC system to avoid damaging the HPLC column; therefore, the real response intensity of the extract would be tens of that shown in Figure 1a. It is also important to note that the gradient elution used in Figure 1a was previously reported for the separation of multiple phenolic compounds [36]. As shown in Figure 1b, this HPLC condition was also suitable for the separation of target neonicotinoid pesticides and the inner standard. Since bee pollen is a rich source of phenolic compounds [39,40], there are complicated peaks in the chromatogram of the SALLE extract; as a consequence, peaks of analytes could not be distinguished from the high background of interferences. This means that the SALLE extract required further clean-up to reduce interfering compounds.

### 2.2. DPX Clean-Up

PSA-based DPX showed a simple, rapid, and efficient clean-up effect for neonicotinoid pesticides in bee pollen. It was interesting to observe that the extract solution became colorless after the PSA-based DPX clean-up. Furthermore, a chromatogram of the cleaned-up solution (Figure 1a) indicated that the intensity of the matrix interferences was dramatically reduced, while a good resolution of analytes with negligible interference was achieved (Figure 1c). These results indicate the excellent clean-up performance of DPX. To reveal the effect of DPX conditions, parameters were systematically investigated.

**Figure 2:**
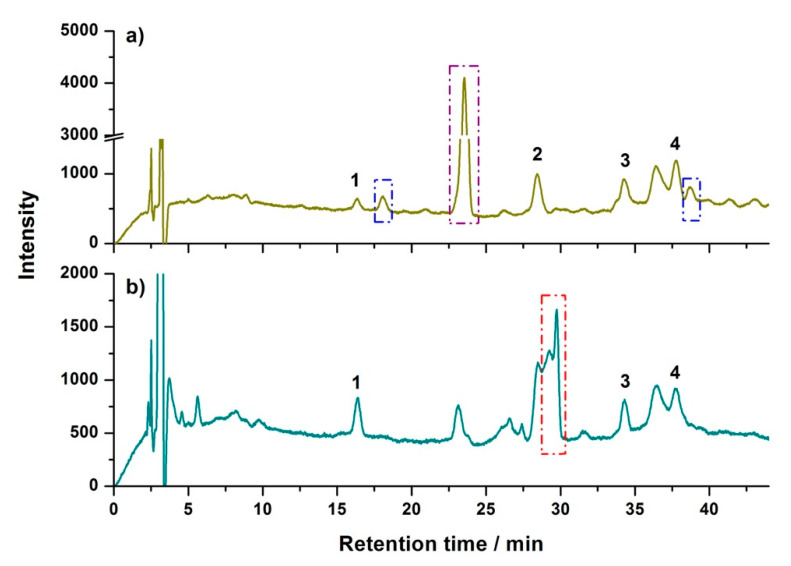
Representative chromatograms of extract cleaned up by (**a**) silica gel DPX, (**b**) dispersive solid-phase extraction (d-SPE). Peak 1: thiamethoxam, 2: acetamiprid, 3: thiacloprid, and 4: ethyl 6-chloropyridine-2-carboxylate (internal standard). The emerging peaks are marked in blue, the high-intensity peak from the matrix is marked in purple, and the matrix peaks overlapped with acetamiprid after d-SPE are marked in red.

Different types of sorbents, including C18 and silica gel, were compared with PSA for clean-up efficiency. In the case of C18, cloudy solutions were obtained after both the loading and the eluting steps. After standing for minutes, sediments were observed in the bottom of the solutions; therefore, the cloudy extract was not further investigated in HPLC. In the silica gel DPX, the final eluent was transparent and colorless; its HPLC chromatogram is shown in Figure 2a. The result indicated that matrix compounds were also dramatically removed by silica gel DPX. Compared with the PSA-based DPX (Figure 1c), the matrix peak located at RT 23.54 min was much higher in silica gel DPX. In addition, peaks emerged at RT 18.08 min and 38.73 min. More importantly, significant leakage of neonicotinoids was observed in the loading step of silica gel DPX, which led to the signal responses of analytes in Figure 2a that were only about 40% of that in PSA-based DPX (Figure 1c). These results indicated that PSA demonstrated better clean-up effect and analytes recovery than silica gel for the analysis of neonicotinoids in bee pollen.

**Figure 3:**
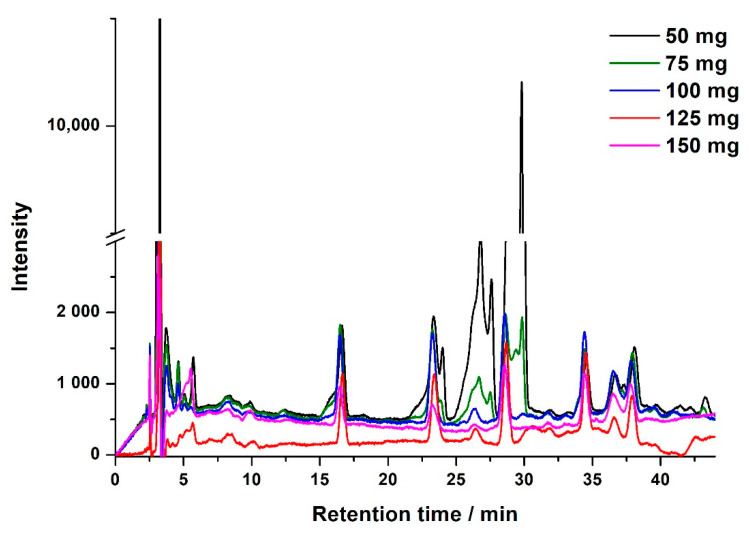
Representative chromatograms of extract cleaned up by DPX tips with different masses of PSA.

The effect of the mass of PSA was studied in the range from 50 mg to 150 mg with increments of 25 mg. It was found that when the mass was from 50 mg to 100 mg, leakage of neonicotinoids was observed in the loading step, and the leaked content was increased with the decreasing of PSA mass. However, leaked analytes were not detected when the mass was further increased to 125 mg and 150 mg. This implies that not less than 125 mg of PSA would be required to prevent the leakage of neonicotinoids in the loading step. Additionally, the mass of PSA also affected the clean-up effect in the following elution step. As shown in Figure 3, in the DPX tips with 50 mg and 75 mg of PSA, peaks of matrix compounds were overlapped with acetamiprid. These interference compounds were eliminated when the mass of PSA was larger than 100 mg. However, as the mass increased to 150 mg, concentrations of neonicotinoids in the eluent were all slightly decreased, which might have resulted from the increasing retention of neonicotinoids in the larger mass of sorbent. Given the absence of analyte leaking in the loading step and its good performance in removing interferences, it was determined that 125 mg of PSA is a suitable choice for the DPX tip.

**Figure 4:**
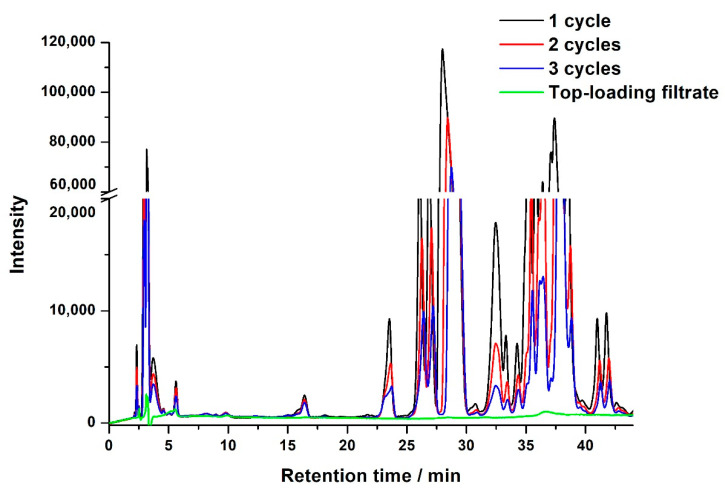
Representative chromatogram of the extract after different in–out cycles using draw-in sample loading, and the filtrate solution after top-loading.

Sample loading was investigated in two modes: draw-in and top-loading. For the draw-in mode, sample solution was aspirated into the DPX column from the bottom of the tip. For the top-loading mode, sample solution was loaded on the top of the DPX tip. In the draw-in mode, increasing the draw-in and dispense-out repeat times (in-out cycles) could improve the clean-up effect. As can be observed in Figure 4, as the in–out cycles increased from 1 to 3 the intensity of matrix compounds decreased significantly. However, the peaks of target compounds were still indistinguishable. In the top-loading mode, the chromatogram of the filtrate solution which was collected by dispensing the loading solution through the DPX tip was very clear and had extremely low levels of matrix compounds (Figure 4). This means that target compounds are all retained on the DPX column; thus, an eluting step is required to wash out the neonicotinoids.

After top-loading the extract, the target compounds were eluted by 100 μL of ACN without a washing step. This elution solvent was also loaded on top of the DPX tip and then dispensed out of the DPX column. Different types of solutions, including ACN, MeOH, H_2_O, and ACN-H_2_O mixture (25%, 50%, 75%, *v*/*v*), were investigated. The MeOH, H_2_O, and ACN-H_2_O mixture all showed the co-elution of matrix interferences. Furthermore, as the H_2_O concentration increased, levels of interference compounds were significantly increased in the eluent. Different volumes of ACN were then compared, and 100 μL was selected as the suitable volume since the further increase in volume would reduce the concentration of neonicotinoids in the eluent, and also increase the risk of the co-elution of matrix interferences.

**Figure 5:**
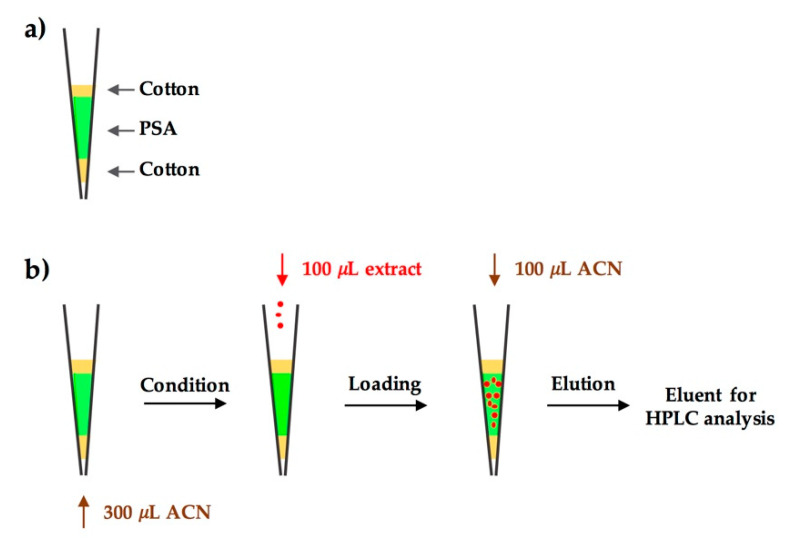
Schematic of (**a**) the DPX device, and (**b**) the optimal DPX procedure.

On the basis of these investigations, the DPX device and the optimal DPX procedure can be proposed, as shown schematically in Figure 5. The DPX column was simply assembled in a 1 mL pipette tip, in which 125 mg of PSA was used as the clean-up sorbent. After being conditioned with 300 μL of ACN solution, 100 μL of SALLE extract was loaded on top of the DPX tip. Then, 100 μL of ACN was applied as the elution solution, which was also added on the top of the pipette tip. The elution was carried out by dispensing the ACN through the DPX column and collecting the eluent for HPLC analysis. The proposed DPX procedure was very simple and rapid; the whole operation could be accomplished in 2 min, using a commercial pipette. Another advantage of the proposed method was the low consumption of sample and solvent, whose total volume was only 500 μL. This DPX method is eco-friendly and may have potential for use in green sample preparation.

Finally, the developed DPX method was compared with the d-SPE procedure [41]. Due to the loss of solution in the d-SPE procedure, 100 μL of SALLE extract was diluted with 300 μL of ACN before being mixed with 125 mg of PSA. As shown in Figure 2b, although the d-SPE procedure also showed significant removal of matrix compounds, its clean-up ability was less efficient than the DPX method, especially for acetamiprid, whose peak was still overlapped with interferences after the d-SPE clean-up.

### 2.3. Analytical Performance

The final cleaned-up solution was separated in C18 reversed-phase HPLC with gradient elution. Target neonicotinoid pesticides were quantified by an inner standard based on our previous report [42]. The diode array detector (DAD) was used for the detection of pesticides, and a wavelength of 254 nm was applied for the quantification. Standards curves were in the linearity range from 0.05 to 3 μg/mL. The detection limit and quantification limit for the analytes were 100 μg/kg and 300 μg/kg with signal to noise of 3 and 10, respectively. The accuracy and precision at two spiked levels (1 × LOQ, 5 × LOQ) are shown in Table 1. Recoveries were between 89.63 and 94.56%, 96.41 and 100.85%, and 86.49 and 96.62% for thiamethoxam, acetamiprid, and thiacloprid, respectively. Intra-day and inter-day precisions were all less than 5%. These results were all in the acceptable range according to the Association of Official Analytical Chemist (AOAC) [43]. Finally, six commercial bee pollen samples were analyzed using the validated method. Results indicated that none of the target neonicotinoid pesticides was detected in these samples. Due to the DPX procedure’s excellent clean-up performance in removing matrix interferences, detection sensitivity towards neonicotinoid pesticides could be significantly improved by combining this simple and rapid DPX method with mass spectrometry detection. These studies are now under way.

## 3. Materials and Methods

### 3.1. Materials

Methanol and acetonitrile (ACN) at HPLC grade were obtained from Merck (Darmstadt, Germany). Acetic acid, anhydrous magnesium sulfate, and sodium chloride at analytical grade were obtained from Sinopharm Chemical Reagent Co., Ltd. (Shanghai, China). Standards of thiamethoxam, acetamiprid, thiacloprid, and ethyl 6-chloropyridine-2-carboxylate (internal standard) were supplied by Aladdin (Shanghai, China). C18 and PSA were from Sepax (Suzhou, China), and silica gel was from Sinopharm. Ultrapure water (18.2 MΩ) was used in all experiments. Rape (*Brassica campestris*) bee pollen used for method development was collected from an apiary in Hubei, China. Commercial rape bee pollen samples used in this application were purchased from local markets. Stock solutions of standards were prepared in ACN with a concentration of 0.2 mg/mL. Working solutions of standards were prepared by further dilution with ACN. All standard solutions were stored at 4 °C until used.

### 3.2. Salting-Out Assisted Liquid-Liquid Extraction

Bee pollen (1 g) was added into 4 mL of ACN-H_2_O solution (1:1, *v*/*v*), then the mixture was homogenized for 30 s by a homogenizer (Fluko, China). After the addition of salts (0.4 g MgSO_4_ and 0.2 g NaCl), the obtained mixture was vortexed for 30 s to dissolve the salts. Then, the final solution was centrifuged at 5000 rpm for 10 min to make phase separation clear. The upper phase was collected for DPX clean-up.

### 3.3. DPX Clean-Up

DPX was assembled in a commercial pipette tip (1 mL). Briefly, PSA (125 mg) was transferred into the pipette tip, with degreasing cotton preloaded at the tip exit for retaining the sorbent. Another pellet of degreasing cotton was then placed on top of the sorbent.

The obtained DPX tip was conditioned with 300 μL of ACN, which was aspirated into the tip from the bottom with an aspiration volume of 800 μL to draw in extra air bubbles for mixing. After bubbling was completed, the solution was dispensed to waste.

Then, 100 μL of SALLE extract was added to the top of the DPX tip and dispensed through the sorbent for sample loading. Finally, 100 μL of ACN was added to the top of the DPX tip and dispensed to elute target compounds. The eluent was collected and transferred into a vial for HPLC analysis.

### 3.4. Study of DPX Procedure

#### 3.4.1. Comparing Matrix Solution without DPX Clean-Up

The SALLE extract (100 μL) was diluted 50 times with the ACN-H_2_O mixture (50%, *v*/*v*) before HPLC analysis.

#### 3.4.2. Effect of Different Sorbents

DPX tips were assembled with 125 mg of C18 or silica gel, and the clean-up was then performed as described in Section 3.3.

#### 3.4.3. Effect of the Mass of PSA

DPX tips were assembled with different masses of PSA (50, 75, 100, 125, and 150 mg). Then, the filtrate solution after sample loading and the final eluent solution as described in Section 3.3 were collected for HPLC analysis.

#### 3.4.4. Effect of Elution Solvent

DPX tips were assembled with 125 mg of PSA. Clean-up was then performed as described in Section 3.3 with different elution solvents, including MeOH, H_2_O, and ACN-H_2_O mixtures (25%, 50%, 75%, *v*/*v*).

#### 3.4.5. Comparing Draw-In Sample Loading Mode

DPX tips assembled with 125 mg of PSA were conditioned as described in Section 3.3. Then, 100 μL of SALLE extract was aspirated into the DPX tip from the bottom with an aspiration volume of 800 μL. After bubbling was completed, the solution was dispensed through the sorbent and collected in another tube. This aspirating in–out operation was repeated three times, and solutions in different cycles were collected for HPLC analysis.

#### 3.4.6. Comparing d-SPE

The d-SPE clean-up experiment was performed on the basis of the reported method [41]. Because of the loss of volume to the sorbent in the d-SPE method, 100 μL of the SALLE extract was diluted with 300 μL of ACN before being mixing with sorbent. The diluted solution was mixed with 125 mg of PSA and vortexed for 1 min. The mixture was then centrifuged at 5000 rpm for 10 min. After centrifugation, the supernatant was collected for HPLC analysis.

### 3.5. HPLC Analysis

HPLC analysis was performed on Shimadzu (Kyoto, Japan) LC-20AT, with a SIL-20AC autosampler, CTO-20AC column oven, and SPD-M20A photodiode array detector. A TSKgel (Tosoh, Japan) ODS-100V column (5 μm, 4.6 × 150 mm) was applied for the separation. The previously reported gradient elution [36,44] was modified for the separation of neonicotinoids. Solvent A was water with 0.1% acetic acid (*v*/*v*), and solvent B was methanol. Elution was as follows: 15% to 40% solvent B at 0–30 min, 40% to 46% solvent B at 30–44 min, post run with 46% to 100% solvent B for 6 min, then back to and maintained at 15% solvent B, each for 5 min. The column temperature was set at 35 °C, the injection volume was 10 μL, the flow rate was 0.8 mL/min, and the detection wavelength was 254 nm.

### 3.6. Method Validation

Seven levels of calibration curves were prepared by standard solutions containing neonicotinoid pesticides (0.05, 0.1, 0.2, 0.5, 1, 2, 3 μg/mL) and IS (2 μg/mL). The ratio of peak area (analyte/IS) versus the ratio of weight (analyte/IS) was used to construct the analytical curves. The y-intercept was set to zero and a linear fit was performed. Limit of detection (LOD) and limit of quantification (LOQ) were investigated in spiked bee pollen samples. Accuracy and precision were estimated by analyzing blank samples spiked at concentrations of 1 × LOQ and 5 × LOQ. Accuracy was expressed as recovery (%), and precision was measured as relative standard deviation (RSD) to the mean recovery of intra-day (*n* = 6) and inter-day (*n* = 18, three days) analyses.

## 4. Conclusions

In summary, a DPX sample preparation method was developed for the HPLC analysis of neonicotinoid pesticides in bee pollen. Effects of the DPX parameters on the clean-up efficiency were systematically investigated. Results revealed that using PSA as the DPX sorbent, combined with top-loading of the sample into the pipette tip and elution, resulted in a remarkable removal of matrix interferences. This PSA-based DPX method exhibited excellent clean-up ability with the merits of simple, rapid, and low solvent consumption. The present work might provide a new strategy for designing fast sample preparation procedures for the challenging and important bee pollen matrix.

## Figures and Tables

**Table 1:** Accuracy and precision of the proposed method at two spiked levels.

Analytes	Spiked Level (μg/kg)	Intra-Day						Inter-Day	
		Day 1		Day 2		Day 3		Mean ± SD (%, *n* = 18)	RSD (%, *n* = 18)
		Mean ± SD (%, *n* = 6)	RSD (%, *n* = 6)	Mean ± SD (%, *n* = 6)	RSD (%, *n* = 6)	Mean ± SD (%, *n* = 6)	RSD (%, *n* = 6)		
Thiamethoxam	300	94.56 ± 4.18	4.42	92.61 ± 3.01	3.25	89.63 ± 2.26	2.52	92.26 ± 3.70	4.01
	1500	92.40 ± 3.46	3.74	91.81 ± 3.68	4.01	89.70 ± 3.46	3.86	91.30 ± 3.53	3.87
Acetamiprid	300	99.13 ± 2.86	2.89	97.82 ± 2.25	2.30	98.18 ± 3.77	3.84	98.38 ± 2.89	2.94
	1500	97.24 ± 3.11	3.20	96.41 ± 2.63	2.73	100.85 ± 3.11	3.08	98.17 ± 3.42	3.48
Thiacloprid	300	86.49 ± 3.81	4.41	86.58 ± 3.36	3.88	87.68 ± 3.31	3.78	86.92 ± 3.49	4.02
	1500	93.29 ± 3.99	4.28	92.96 ± 3.38	3.64	96.62 ± 1.95	2.02	94.29 ± 3.47	3.68

## Supplementary Materials

The following are available online, Figure S1: Structures and LogP values of analytes.
