# Supplementary material for: Miniaturized Salting-Out Assisted Liquid-Liquid Extraction Combined with Disposable Pipette Extraction for Fast Sample Preparation of Neonicotinoid Pesticides in Bee Pollen
Source: Molecules. 2020 Dec 3;25(23):5703. doi: 10.3390/molecules25235703 (PMC7729831; doi:10.3390/molecules25235703)
Supplement: Supplementary file 1 [file molecules-25-05703-s001.pdf]

molecules

MDPI

Supplementary material

Miniaturized salting-out assisted liquid-liquid extraction combined with disposable pipette extraction for fast sample preparation of neonicotinoid pesticides in bee pollen

Xijuan Tu [1,2], and Wenbin Chen [1,2,*]

[1] College of Bee Science, Fujian Agriculture and Forestry University, Fuzhou 350002, China.
[2] College of Animal Science, Fujian Agriculture and Forestry University, Fuzhou 350002, China.
* Correspondence: wbchen@fafu.edu.cn

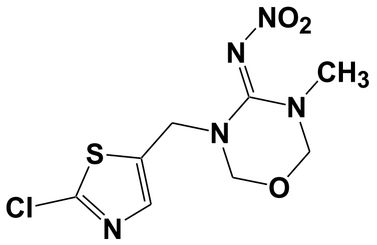

Thiamethoxam, LogP: -1.16

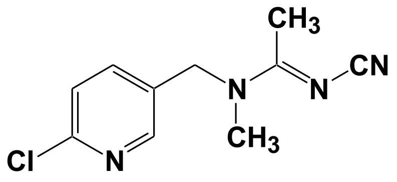

Acetamiprid, LogP: 0.62

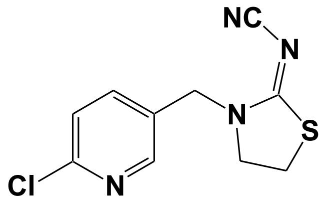

Thiacloprid, LogP: 0.55

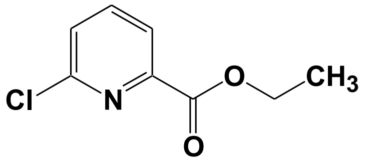

Ethyl 6-chloropyridine-2-carboxylate, LogP: 1.77

Figure S1. Structures and LogP values of the investigated neonicotinoid pesticides (thiamethoxam, acetamiprid, and thiacloprid), and the internal standard (ethyl 6-chloropyridine-2-carboxylate). The LogP values were obtained from ChemSpider [1].

Reference:

1. ChemSpider, http://www.chemspider.com (accessed on 28 November 2020).
